# Inflammatory pathways in heart failure with preserved left ventricular ejection fraction: implications for future interventions

**DOI:** 10.1093/cvr/cvac133

**Published:** 2022-08-25

**Authors:** Nicola Riccardo Pugliese, Pierpaolo Pellicori, Francesco Filidei, Nicolò De Biase, Pasquale Maffia, Tomasz J Guzik, Stefano Masi, Stefano Taddei, John G F Cleland

**Affiliations:** Department of Clinical and Experimental Medicine, University of Pisa, Pisa 56126, Italy; Robertson Institute of Biostatistics and Clinical Trials Unit, University of Glasgow, Glasgow G12 8QQ, UK; Department of Clinical and Experimental Medicine, University of Pisa, Pisa 56126, Italy; Department of Clinical and Experimental Medicine, University of Pisa, Pisa 56126, Italy; Centre for Immunobiology, Institute of Infection, Immunity and Inflammation, College of Medical, Veterinary and Life Sciences, University of Glasgow, Glasgow G12 8TA, UK; Institute of Cardiovascular and Medical Sciences, College of Medical, Veterinary and Life Sciences, University of Glasgow, Glasgow G12 8QQ, UK; Department of Pharmacy, School of Medicine and Surgery, University of Naples Federico II, Naples 80138, Italy; Institute of Cardiovascular and Medical Sciences, College of Medical, Veterinary and Life Sciences, University of Glasgow, Glasgow G12 8QQ, UK; Department of Internal and Agricultural Medicine, Jagiellonian University, Collegium Medicum, Krakow 31-008, Poland; Department of Clinical and Experimental Medicine, University of Pisa, Pisa 56126, Italy; Department of Clinical and Experimental Medicine, University of Pisa, Pisa 56126, Italy; Robertson Institute of Biostatistics and Clinical Trials Unit, University of Glasgow, Glasgow G12 8QQ, UK

**Keywords:** Epidemiology, Hypertension, Global, International, Cardiovascular

## Abstract

Many patients with symptoms and signs of heart failure have a left ventricular ejection fraction ≥50%, termed heart failure with preserved ejection fraction (HFpEF). HFpEF is a heterogeneous syndrome mainly affecting older people who have many other cardiac and non-cardiac conditions that often cast doubt on the origin of symptoms, such as breathlessness, or signs, such as peripheral oedema, rendering them neither sensitive nor specific to the diagnosis of HFpEF. Currently, management of HFpEF is mainly directed at controlling symptoms and treating comorbid conditions such as hypertension, atrial fibrillation, anaemia, and coronary artery disease.

HFpEF is also characterized by a persistent increase in inflammatory biomarkers. Inflammation may be a key driver of the development and progression of HFpEF and many of its associated comorbidities. Detailed characterization of specific inflammatory pathways may provide insights into the pathophysiology of HFpEF and guide its future management. There is growing interest in novel therapies specifically designed to target deregulated inflammation in many therapeutic areas, including cardiovascular disease. However, large-scale clinical trials investigating the effectiveness of anti-inflammatory treatments in HFpEF are still lacking. In this manuscript, we review the role of inflammation in HFpEF and the possible implications for future trials.

## Graphical Abstract

**Graphical Abstract:**
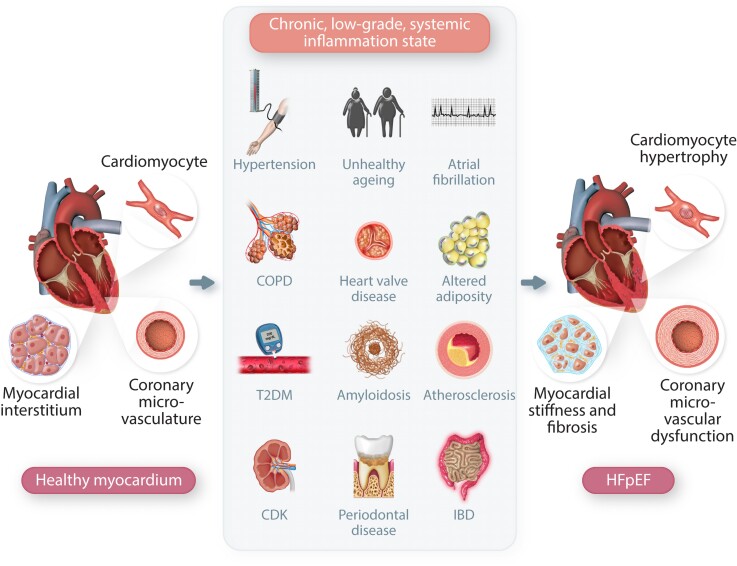
The comorbidity-inflammation paradigm in heart failure with preserved ejection fraction (HFpEF). Unhealthy ageing and highly prevalent comorbidities induce a chronic, low-grade, systemic inflammatory state, which drives the progression from healthy myocardium towards HFpEF. CKD, chronic kidney disease; CMD, coronary microvascular dysfunction; COPD, chronic obstructive pulmonary disease; HFpEF, heart failure with preserved ejection fraction; IBD, inflammatory bowel disease; T2DM, Type 2 diabetes mellitus.

**This manuscript was handled by Guest Editor Carolyn S.P. Lam.**

**This article is part of the Spotlight Issue on Heart Failure.**

## 1. Introduction

Many patients diagnosed with heart failure (HF) have a left ventricular ejection fraction (LVEF) ≥50%, i.e. HF with preserved LVEF (HFpEF).^1^ Nevertheless, making a diagnosis of HFpEF is challenging. Most patients are elderly, with a high proportion of women and several cardiovascular and non-cardiovascular comorbidities.^1^ Outpatients with HFpEF have a better overall prognosis and a much lower rate of cardiovascular events than those with HF with reduced LVEF (HFrEF), but a higher proportion of non-cardiovascular deaths.^2^ However, among patients with decompensated HF, the outcome is similar for HFpEF and HFrEF, although the prognosis for HFpEF might often be driven by comorbid disease rather than HF itself.^3^ Also, HFpEF and HFrEF are not entirely distinct entities. Indeed, impaired myocardial contraction is expected in both, e.g. circumferential for HFrEF and long axis for HFpEF.^4,5^ However, measurement of LVEF is prone to substantial error—hence the introduction of the HFmrEF phenotype, which acts as a buffer zone to reduce classification error between HFrEF and HFpEF in research and clinical practice. HFrEF may recover, especially for younger patients with little myocardial scar (e.g. dilated cardiomyopathy) who receive guideline-recommended therapy; this should not be considered HFpEF but rather HF with recovered LVEF.^6,7^ For patients with HFpEF, LVEF may also drop over time due to measurement error [increased with the onset of atrial fibrillation (AF)]^8^ or additional myocardial insults (e.g. myocardial infarction).^5^

A series of randomized control trials (RCTs) of neurohormonal antagonists failed to show clear cardiovascular benefits in patients with HFpEF.^1^ Recently, a RCT on a factor Xa antagonist demonstrated a substantial reduction in mortality for patients with HFpEF and coronary artery disease (CAD),^9^ while two others found that sodium-glucose cotransporter-2 inhibitors (SGLT2i) reduce hospitalizations for worsening HF.^10,11^ These trials showing that the natural history of HFpEF can be favourably modified encourage exploring other therapeutic avenues, including inflammation, which might have a key role in the pathophysiology underlying HFpEF.^12^ A thorough characterization of inflammatory pathways involved in HFpEF might help identify therapeutic targets and interventions. Herein, we provide an overview of the role of inflammation in the pathogenesis of HFpEF, summarize the available evidence for anti-inflammatory treatments, and discuss the potential implications for the design of future HFpEF trials. We deliberately avoid a detailed discussion of antifibrotic therapies because fibrosis is a non-specific downstream consequence of myocardial damage.^12,13^

## 2. The inflammatory-metabolic phenotype and HFpEF

Inflammatory biomarkers, including tumour necrosis factor (TNF)-α and its receptors (TNFR1 and TNFR2), interleukin (IL)-6 and IL-8, high-sensitivity C-reactive protein (hsCRP), pentraxin-3 and the chemokine (C–C motif) ligand 2 (CCL2), also referred to as monocyte chemoattractant protein-1 (MCP-1), are often elevated in patients with HFpEF.^14,15^ Chronic, low-grade, systemic inflammation might have detrimental effects on myocardial structure and function (*Graphical Abstract*). Experimental models suggest that increased production of pro-inflammatory cytokines enhances oxidative stress, drives the differentiation of fibroblasts into collagen-secreting myofibroblasts, and induces extracellular matrix degradation, leading to increased myocardial stiffness and coronary microvascular dysfunction (CMD).^16,17^ Local inflammation also reduces nitric oxide (NO) and cyclic guanosine monophosphate (cGMP) availability, resulting in the hypo-phosphorylation of the giant sarcomeric protein titin, which further increases myocardial stiffness and worsens diastolic function.^16^ Oxidative stress might also be implicated in the development of metabolic heart disease, indicating bidirectional links between inflammation and cardiac dysfunction.^18^

Whether the persistent inflammation that characterizes HFpEF represents a causal factor or an epiphenomenon due to one or more pro-inflammatory comorbid conditions is not well understood.^16^ Indeed, inflammation is frequently associated with unhealthy ageing and several cardio-metabolic comorbidities (*Graphical Abstract*), e.g. obesity and altered adiposity, chronic kidney disease (CKD), hypertension, CAD, Type 2 diabetes mellitus (T2DM), AF, elevated serum uric acid (SUA) concentrations, and chronic obstructive pulmonary disease (COPD).^19–22^ Chronic low-grade inflammation might also contribute to the development of sarcopaenia and frailty, which are very common among outpatients with HFpEF, with a prevalence varying from 30 to 52%.^23^ All these features, particularly when combined, might lead to a poorer quality of life and drive cardiovascular and non-cardiovascular outcomes.^24,25^ Recently, proteomic bioprofiles have been investigated to identify potential mechanistic pathways associated with HF development. Systemic inflammation might mediate the association between several comorbidities and cardiac dysfunction, promoting the progression of disease.^25–27^

AF and HFpEF frequently co-exist. Chronically elevated left atrial pressures might produce adverse structural atrial remodelling and dysfunction that increase the risk of developing AF. On the other hand, AF, especially with a rapid ventricular response, leads to a decreased LV filling time and the loss of atrial contribution to LV filling; therefore, it might precipitate the onset or worsening of HFpEF.^28^ Biomarkers reflecting systemic congestion, inflammation, and fibrosis predict clinically overt AF as well as HFpEF and are associated with abnormal diastolic filling and reduced exercise capacity.^29^ Endothelial inflammation can cause CMD and myocardial fibrosis, which can induce both atrial and ventricular myopathy.^30^ In addition, systemic inflammatory and metabolic disorders have been linked to an expansion and pro-inflammatory transformation of epicardial adipose tissue (EAT)^31–35^ EAT accumulation may also impair atrial distension and contraction by its proximity to the myocardium, leading to mechanical dysfunction, electroanatomical fragmentation, and ultimately AF.^36,37^

Patients with HFpEF may exhibit different inflammation patterns according to their comorbidities. Inflammation in hypertension is often driven by renin–angiotensin–aldosterone system activation.^38,39^ On the other hand, the inflammatory milieu of obese patients is primarily due to adipokines, i.e. cytokines secreted by both adipocytes and macrophages resident in adipose tissue.^40^ Patients with HFpEF who are obese have higher serum concentrations of many pro-inflammatory proteins (galectin-9, CD4, and TNF-related apoptosis-inducing ligand receptor 2).^41^ This heterogeneity in inflammatory phenotypes could lead to the application of precision medicine in HF treatment, with different therapeutic approaches according to the pattern of comorbidity.^42^

Other non-metabolic diseases cause a chronic, low-grade, systemic inflammatory response, and produce a clinical picture that fits the HFpEF definition. Amyloidosis is a systemic disorder characterized by the progressive deposition of fibrillary proteins that cause immune cell infiltration into tissues and pro-inflammatory cytokine production in various organs, eventually resulting in their failure.^43^ Amyloid deposition might be common in patients with HFpEF,^44^ but, until recently, the diagnosis of amyloidosis has seldom been considered and rarely investigated. Heart valve diseases, even if not severe, can worsen cardiac structure and function and contribute to HF symptoms in the presence of a normal LVEF: it is noteworthy that degenerative valve disease is the most frequent valvular disorder in Western countries and is characterized by calcification, which might, in turn, be related to inflammation.^45^ Chronic inflammatory disorders, such as inflammatory bowel disease and rheumatoid arthritis, have been linked to an increased risk of cardiovascular disease (CVD) and HF, especially during flares in disease activity.^46,47^ Periodontal disease is extremely common and associated with increased cardiovascular risk.^48^ The more severe the periodontitis, the higher the risk of developing HF; conversely, good oral hygiene reduces it.^49,50^

## 3. How to identify and quantify inflammation in clinical practice

### 3.1 Inflammatory biomarkers

The inflammatory profile of patients with HFpEF can be investigated by measuring plasma concentrations of inflammatory biomarkers. However, plasma concentrations reflect a steady-state between production and disposal and are not necessarily evidence of changes in inflammation. In the presence of renal or hepatic dysfunction, elevated plasma concentrations may reflect reduced clearance rather than increased production.^51^

**Figure 1:**
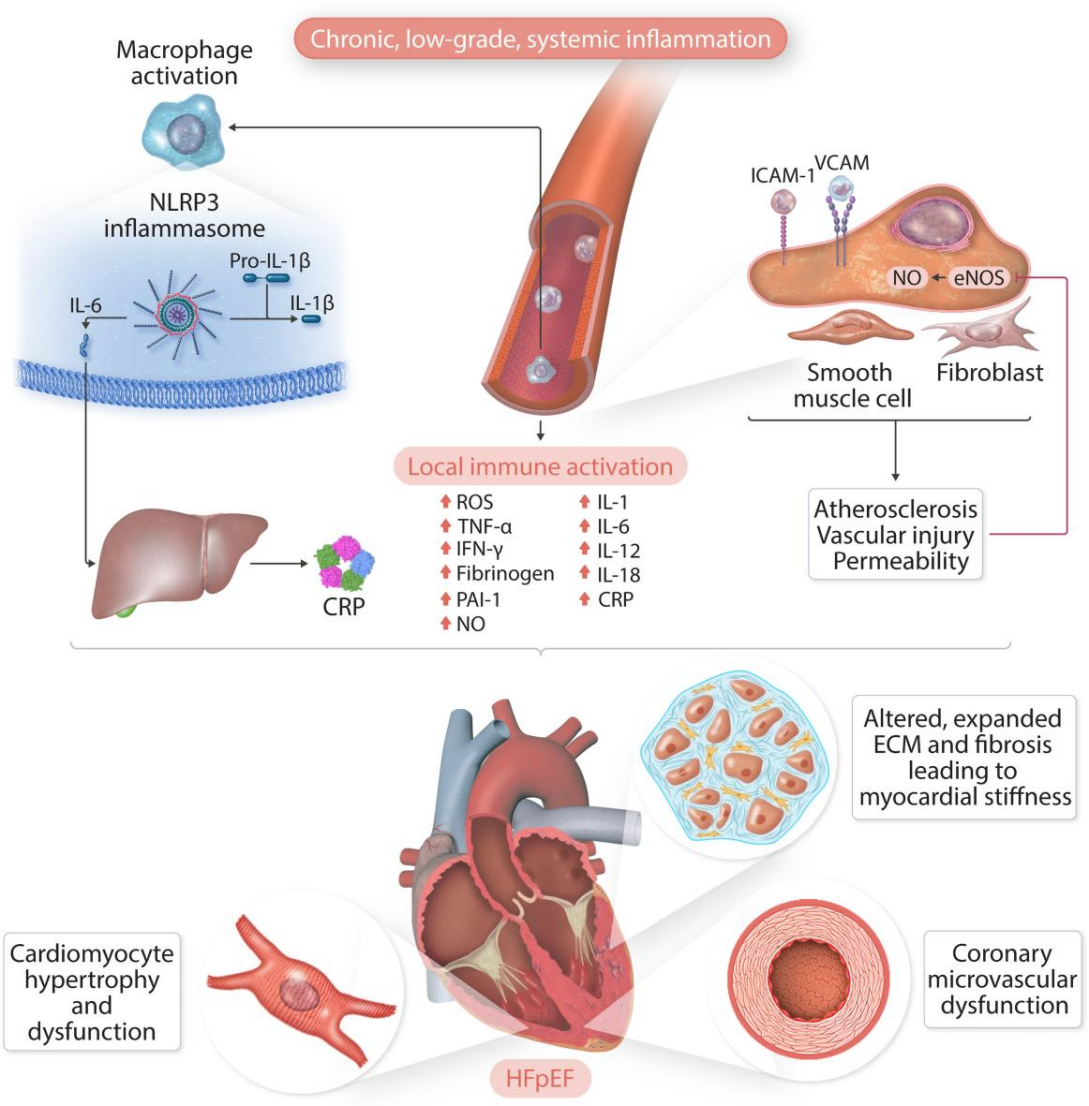
The inflammatory pathways. Chronic, low-grade systemic inflammation activates nucleotide oligomerization domain-like receptor family, pyrin domain-containing (NLRP3) inflammasome, leading to the cleavage and activation of pro-inflammatory cytokines. Inflammation also promotes endothelial dysfunction, atherosclerosis, and vascular injury. All these alterations may contribute to the development of heart failure with preserved ejection fraction (HFpEF). CMD, coronary microvascular dysfunction; eNOS, endothelial nitric oxide synthase; ICAM-1, intercellular adhesion molecule-1; IL-1β, interleukin-1β; IL-6, interleukin-6; NO, nitric oxide; PAI-1, plasminogen activator inhibitor-1; TNF-a, tumour necrosis factor-a; VCAM, vascular cell adhesion molecule; VSMC, vascular smooth muscle cell.

The most explored inflammatory pathway involves the nucleotide oligomerization domain-like receptor family, pyrin domain-containing (NLRP3) inflammasome (*Figure 1*), with the subsequent cleavage and activation of IL-1β, IL-6, IL-12, IL-18 and, finally, the production of CRP in the liver.^52^ This pathway is driven by endogenous stimuli and defined as ‘sterile’ inflammation instead of exogenous-induced inflammation due to infection. In routine clinical practice, hsCRP is widely available, relatively stable in peripheral blood and the most commonly measured inflammatory biomarker. High concentrations of hsCRP are associated with more comorbidity and greater disease severity in HFpEF and HFrEF,^53,54^ and predict a worse outcome, although for HFpEF this is mainly for non-cardiovascular events.^53^

In several population-based longitudinal registries including patients with cardiovascular risk factors, SUA levels are associated with an increased risk of all-cause and cardiovascular mortality at serum concentrations substantially lower than those used to define hyperuricaemia in clinical practice.^55,56^ One potential explanation for this association is the induction of systemic inflammation due to oxygen-free radical production during the conversion of purines to uric acid by xanthine oxidase.^22,57^ The ESC-EORP-HF-LT registry showed that higher SUA concentrations are associated with an adverse prognosis for both HFrEF and HFpEF.^58^ However, uric acid itself is a powerful antioxidant,^59^ which may account for why randomized trials of xanthine oxidase inhibitors have, so far, failed to show convincing cardiovascular benefits.^60^ A large randomized trial of allopurinol for patients with ischaemic heart disease (IHD) should report soon.^61^

Galectin-3 is a lectin expressed by activated cardiac macrophages and induces the secretion of IL-6. Elevated serum concentrations of galectin-3 are associated with adverse LV remodelling, cardiomyocyte hypertrophy, and myocardial fibrosis.^62^ In the Aldo-DHF trial, baseline serum concentrations of galectin-3 were inversely correlated with functional capacity and directly associated with NYHA class in HFpEF patients.^62^ Data from multiple RCTs and registries suggest that galectin-3 is an independent predictor of adverse outcomes in chronic and acutely decompensated HF, regardless of LVEF.^62,63^ Thus, galectin-3 has been proposed as a marker of fibrotic activity,^64^ potentially mediated by inflammation. However, it is excreted by the kidney, and not all studies have corrected for renal function.

High serum concentrations of the soluble suppression of tumourigenicity 2 (sST2, the circulating form of the cellular ST2 receptor, expressed by cardiac and vascular cells together with its ligand IL-33 following cardiovascular injury) are also associated with increased myocardial fibrosis and inflammation as well as with poorer outcomes in patients HFpEF.^65–67^ Many other markers of inflammation, metabolic dysfunction, and extracellular matrix remodelling (e.g. tissue inhibitors of metalloproteinases, procollagen Type I C-terminal propeptide, and procollagen Type III N-terminal propeptide (PIIINP), adrenomedullin, cystatin C, and resistin) have prognostic significance in HFpEF^68–70^ but have yet to find a role in clinical practice.

Ferritin binds to iron in cells. In the absence of other diseases, iron deficiency is associated with low release of ferritin, and low serum concentrations signify iron deficiency. However, in the presence of inflammation, serum ferritin leaks from cells rendering it unreliable as a marker of iron deficiency, which might mask a diagnosis of iron deficiency in HFpEF.^71^ Iron deficiency is common in HF, irrespective of LVEF and associated with adverse outcomes.^72^ Inflammation can also inhibit iron absorption and iron mobilization and/or utilization even when iron stores are not depleted. Pro-inflammatory cytokines (e.g. TNF-α, IL-1β, IL-6) upregulate the protein hepcidin, leading to reduced intestinal iron absorption and decreased iron mobilization from bone marrow stores.^73^ Intravenous iron administration in patients with systolic HF improves symptoms and clinical outcomes, but evidence of benefit is still lacking for those with HFpEF.^74^

Omics techniques such as single-cell RNA sequencing are an emerging tool for studying the transcriptional heterogeneity in both healthy and diseased hearts^75^ and the diversity of immune cells implicated in the development of CVD.^76^ Multi-omics approaches may soon uncover novel inflammatory cardiac pathways that offer new therapeutic opportunities.

### 3.2 Imaging techniques

Echocardiography is the most widely available imaging technique and provides valuable information regarding structural and functional alterations in HFpEF. The *E*/*e*’ ratio is a widely used surrogate marker of LV filling pressures, but its clinical utility remains controversial, and it has not been used to select patients for any of the successful landmark trials of HFpEF.^77^ Left atrial volume and function might be the best marker to integrate left ventricular systolic and diastolic dysfunction, including the mitral apparatus and heart rhythm^78,79^ but will be agnostic to the underlying pathophysiology. Echocardiography can also evaluate EAT accumulation,^80^ which is associated with greater inflammatory activity, impaired haemodynamics, worse symptoms, and a poorer prognosis in patients with HF.^81^

Echocardiography and, more generally, ultrasound represent a valuable tool to estimate congestion,^82^ which is central to the diagnosis and prognosis of HF and is also associated with increased inflammatory activity.^83^ In experimental conditions, the development of venous congestion activates the innate immune system and the secretion of pro-inflammatory cytokines in healthy individuals.^84^ Therefore, treatment targeted at congestion might improve inflammation. Intensification of therapy with loop diuretics had a mixed effect on biomarkers of immune activation and inflammation in one small study of HFrEF, but it normalized endotoxin, which is often increased in congestive HF due to altered gut permeability and subsequent translocation of lipopolysaccharide into the circulation.^85,86^

Cardiac magnetic resonance (CMR) represents the gold standard for evaluating chamber geometry and structure, as well as quantifying EAT volume with great precision.^87,88^ CMR can also be used to estimate the extracellular volume and myocardial oedema.^89^ In HFpEF, the former correlates with myocardial stiffness and the extent of interstitial collagen deposition evaluated at the histopathological level,^89^ while the latter could be related to increased microvascular permeability or impaired lymphatic function.^90^

Localized myocardial inflammation can be assessed by positron emission tomography using [18F]fluorodeoxyglucose, which accumulates in activated inflammatory cells (monocyte, macrophage, lymphocyte) due to increased glucose uptake. Mechanistic studies suggest that [18F]fluorodeoxyglucose imaging after myocardial infarction (MI) and pressure-overload HF may provide additional prognostic information.^91^

## 4. Inflammation as a therapeutic target in clinical trials of HFpEF

Many large RCTs have investigated the effect of anti-inflammatory agents on cardiovascular events in patients with atherosclerosis, hypertension, and other cardiovascular disorders (*Table 1*).^92^ Most of these trials excluded patients with moderate or severe HF, did not always include HF-related endpoints and often did not provide data on LVEF,^93,95–100,102,104,105^ limiting extrapolation of their results to patients with HFpEF. Nevertheless, IHD is frequently undiagnosed and sub-optimally treated in patients with HFpEF.^1^ Not many patients with HFpEF undergo coronary angiography due to their advanced age, the high number of comorbidities, particularly renal dysfunction, and the lack of evidence that revascularization is beneficial in the absence of acute ischaemia. Of those who are investigated, many (>50%) have obstructive epicardial CAD, and most (85%) have evidence of CMD.^111^ The presence of CAD is not surprising, given the high proportion of patients with HFpEF who have hypertension, T2DM, obesity, and CKD. These comorbidities also account for the high prevalence of CMD, which may play a key pathophysiological role in the development of HFpEF, independent of atherosclerotic burden.^112,113^ Inflammation promotes all stages of atherosclerosis, from plaque formation to rupture, leading to macrovascular and microvascular ischaemic events.^76,114^ Also, chronic inflammation, even in the absence of epicardial stenoses and traditional coronary risk factors, is associated with CMD.^115^

**Table 1:** Clinical trials investigating anti-inflammatory agents in ischaemic heart disease

Trial (year)	Setting	Intervention	No. of Patients	FU (months)	Primary endpoint	Results	Study limitations
*Antioxidants*							
ARISE (2008)^93^	Recent ACS; HF: 15%	Succinobucol vs. placebo	6144	24	CVM, RCA, MI, CVA, UA, or Revasc.	No difference.	No data on LVEF.
*Immunosuppressive agents*							
METIS (2009)^94^	IHD; HF: 100% LVEF: ∼35%	Methotrexate vs. placebo	50	3	Difference in 6MWT.	No difference.	Small trial Median hs-CRP at baseline only 2.8 mg/L.
CIRT (2019)^95^	IHD with T2DM or metabolic syndrome; HF: 13%.	Methotrexate vs. placebo	4786	28	Non-fatal MI, RCA, CVM, or urgent Revasc.	No difference. Safety: ↑ cancer (mostly skin basal-cell) with methotrexate.	No data on LVEF.
*Colchicine*							
LoDoCo^a^ (2013)^96^	Chronic CAD	Colchicine vs. standard care	532	36	ACS, RCA, or ischaemic CVA.	5.3% on colchicine vs. 16.0% with standard care (HR: 0.33, 0.18–0.59; *P* < 0.001).	History of HF not reported.
COLCOT^a^ (2019)^97^	Recent MI; HF: 1.9%	Colchicine vs. placebo	4745	23	CVM, RCA, MI, CVA, or urgent Revasc.	5.5% on colchicine vs. 7.1% on placebo (HR: 0.77, 0.61–0.96; *P* = 0.02). Safety: more pneumonia with colchicine v. placebo 0.9% vs. 0.4%, *P* = 0.03).	Relatively short follow-up. No data on LVEF.
LoDoCo2^a^ (2020)^98^	Chronic CAD (severe HF excluded)	Colchicine vs. placebo	5522	29	CVM, MI, ischaemic CVA, or urgent Revasc.	6.8% on colchicine vs. 9.6% on placebo (HR: 0.69, 0.57–0.83; *P* < 0.001).	History of HF not reported. No baseline CRP.
COPS (2020)^99^	ACS and evidence of CAD managed with PCI or medical therapy.	Colchicine vs. placebo	795	12	All-cause mortality, ACS, urgent Revasc, or ischaemic CVA.	No difference. Safety concern: ↑ACM (8 vs. 1, *P* = 0.017) and non-CVM with colchicine (5 vs. 0, *P* = 0.024).	History of HF not reported.
*Phospholipase A_2_ inhibitors*							
SOLID-TIMI 52 (2014)^100^	Recent ACS (NYHA III–IV HF excluded).	Darapladib vs. placebo	13 026	30	CAD-related death, MI, or urgent Revasc.	No difference.	History of HF not reported. No data on LVEF.
STABILITY (2014)^101^	Chronic CAD (NYHA III–IV HF excluded).	Darapladib vs. placebo	15 828	444	CVM, MI, or CVA.	No difference.	History of HF not reported. No LVEF data
VISTA-16 (2014)^102^	ACS; HF: 17.8%.	Varespladib vs. placebo	5145	6	CVM, MI, CVA, or UA.	No difference.	Termination of the trial for futility and possible harm. No LVEF data.
*Cholesterylester transfer protein inhibitors*							
Dal-GenE (2022)^103^	Recent ACS and the AA genotype at variant rs1967309 in the ADCY9 gene.	Dalcetrapib or placebo	6149	39.9	CVM, RCA, non-fatal MI, and non-fatal stroke.	No difference.	COVID-19 pandemic during study conduct.
*Anti-IL-1*							
MRC-ILA Heart study^a^ (2015)^104^	Recent NSTE-ACS.	Anakinra vs. placebo	182	12	AUC for CRP over the first 7 days.	AUC for CRP ↓ with anakinra vs. placebo (*P* = 0.003). With IL-1ra, at 14 days ↓ hs-CRP (*P* < 0.0001) and ↓ IL-6 (*P* = 0.02).	History of HF not reported. Small sample size.
CANTOS^a^ (2017)^105,106^	Prior MI and hs-CRP ≥2 mg/L; HF: 22%.	Canakinumab (three different doses) vs. placebo	10 061	444	MI, CVA, or CVM.	50 mg dose, no difference vs. placebo; 150 mg dose (0.85: 0.74–0.98, *P* = 0.021); 300 mg dose (0.86: 0.75–0.99, *P* = 0.03). Safety: Canakinumab associated with ↑ of fatal infection but ↓ (lung) cancer.	No data on LVEF.
VCU-ART 3^a^ (2020)^107^	STEMI within 12 h of symptom onset (mean LVEF: 51%).	Anakinra vs. placebo	99	12	AUC for hs-CRP after 14 days.	AUC for CRP ↓ with Anakinra vs. placebo (*P* < 0.001).	History of HF not reported. Small sample size. Missing data.
*Anticoagulation*							
COMMANDER-HF	Chronic HFrEF (LVEF ≤40%), CAD and sinus rhtythm, recently hospitalized for HF.	Rivaroxaban 2.5 mg bid vs. placebo	5022	21	ACM, MI, or CVA.	No difference.	Only HFrEF.
COMPASS pre-planned subanalysis^a^ (2019)^9^	CAD or peripheral artery disease; HF: 22%.	Rivaroxaban 2.5 mg bid + ASA 100 mg and rivaroxaban 5 mg bid alone, vs. ASA 100 mg alone	27 395	23	CVM, CVA, or MI.	Rivaroxaban + ASA reduced endpoint in patients without (0.79: 0.68–0.93) and with HF (0.68: 0.53–0.86, *P* for interaction 0.28) with larger absolute risk reduction in those with HF (2.4 vs. 1.0%) vs. ASA alone. No significant differences with rivaroxaban alone.	Only 84% of HF patients had LVEF recorded at baseline (only 12% had LVEF <40%).
*Influenza vaccination*							
IAMI^a^ (2021)^108^	Recent ACS; Acute HF: 3.8%.	Influenza vaccine vs. placebo	2532	12	ACM, MI, or stent thrombosis.	5.3% after influenza vaccine vs. 7.2% with placebo (0.72: 0.52–0.99, *P* = 0.04). ACM: 2.9 vs. 4.9% (0.59: 0.39–0.89, *P* = 0.01). CVD: 2.7 vs. 4.5% (0.59: 0.39–0.90, *P* = 0.014). MI: 2.0 vs. 2.4% (0.86: 0.50–1.46, *P* = 0.57). Stent thrombosis: 0.5 vs. 0.2% (1.94: 0.48–7.76, *P* = 0.34).	History of HF not reported. LVEF ≥50% at discharge in 60.5% of partecipants.
IVVE^109^ (2022)	Chronic HF in low and/or middle income countries.	Influenza vaccine vs. placebo	2569	36	CVM, non-fatal MI, non-fatal stroke.	No difference for primary. All hospitalizations and pneumonia were reduced. Reductions in the primary endpoint were noted during peak influenza season.	
*Epigenetic regulators*							
BETonMACE pre-planned subanalysis^a^ (2021)^110^	T2DM up to 3 months after ACS.	Apabetalone vs. placebo	2425	26	Hosp. for HF.	Apatabetalone ↓ first HF hosp.: 2.4 vs. 4.0% (0.59: 0.38–0.94, *P* = 0.03), total HF hosps.: 35 vs. 70 (0.47: 0.27–0.83, *P* = 0.01), and the composite of CVM or HF hosp.: 5.7 vs. 7.8% (0.72: 0.53–0.98, *P* = 0.04).	No data on LVEF.

Results are presented as (hazard ratio: 95% confidence interval, *P*-value). ACM, all-cause mortality; ACS, acute coronary syndrome; ASA, acetylsalicylic acid; AUC, area under the curve; CAD, coronary artery disease; CI, confidence interval; CK-MB, creatin kinase-muscle brain; CMR, cardiac magnetic resonance; CVA, cerebrovascular accident; CVM, cardiovascular mortality; EF, ejection fraction; HDL-C, high-density lipoprotein-cholesterol; HF, heart failure; HR, hazard ratio; hs-CRP, high-sensitivity C-reactive protein; IL-6, interleukin-6; Lp-PLA_2_, lipoprotein-associated phospholipase A_2_; LVEF, left ventricular ejection fraction; LVESV, left ventricular end-systolic volume; MACE, major adverse cardiovascular events; MI, myocardial infarction; NSTE, non-ST elevation; PCI, percutaneous coronary intervention; PROBE, prospective randomized observer-blinded endpoint; RCA, resuscitated cardiac arrest; Revasc, coronary revascularization; sPLA_2_, soluble phospholipase A_2_; STEMI, ST-elevation myocardial infarction; SVG, saphenous vein graft; T2DM, Type 2 diabetes mellitus; TnI, troponin I; UA, unstable angina.

The trial met the primary endpoints.

**Figure 2:**
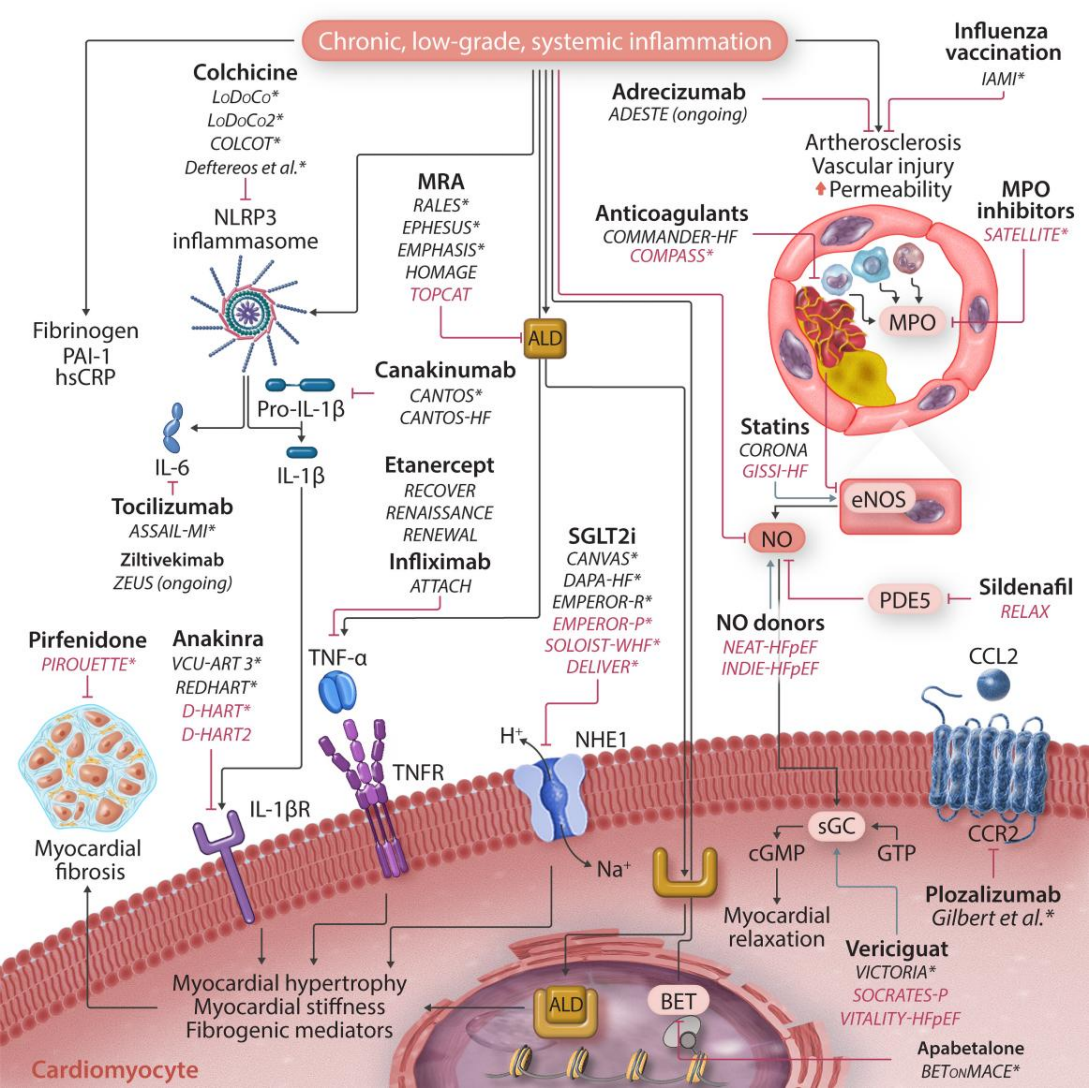
Clinical trials targeting inflammatory pathways in patients with heart failure. Many different biochemical pathways are involved in inflammation-driven heart injury and can be targeted at different levels. Drugs are in bold, while clinical trials are in italics (trials that included patients with HFpEF are marked blue). ‘*’ denotes trials/studies that met their primary endpoint. BET, bromodomain and extra-terminal motif; CCL2, C–C chemokine ligand 2; CCR2, C–C chemokine receptor Type 2; cGMP, cyclic guanosine monophosphate; ECM, extracellular matrix; eNOS, endothelial nitric oxide synthase; GTP, guanosine triphosphate; HFpEF, heart failure with preserved ejection fraction; IL-1β-R, interleukin-1β receptor; MPO, myeloperoxidase; NLRP3, nucleotide oligomerization domain-like receptor family, pyrin domain-containing; NO, nitric oxide; PDE5, phosphodiesterase-5; sGC, soluble guanylate cyclase; TNFR, tumour necrosis factor receptor.

Inflammation has been the therapeutic target for many RCTs that enrolled only patients with HF (*Table 2* and *Figure 2*), but, until now, most of them were focused on the HFrEF phenotype.^116–118^

**Table 2:** Clinical trials investigating anti-inflammatory agents in heart failure

Trial (year)	Setting	Treatment groups	No. of patients	Follow-up	Primary endpoint	Results	Study limitations
*Anti-TNF-α*							
ATTACH (2003)^116^	Chronic HF (NYHA III–IV, LVEF ≤35%)	Infliximab vs. placebo	150	6	Change in clinical status at 14 weeks.	No difference.	Only HFrEF
RENAISSANCE, RECOVER, RENEWAL (2004)^117^	Chronic HF (NYHA II–IV, LVEF ≤30%)	Etanercept vs. placebo	2048	6	RENAISSANCE and RECOVER: clinical status at 24 weeks. RENEWAL: CVM or HF hosp. from RENAISSANCE and RECOVER.	All neutrals. Safety: more infections with etanercept.	Only HFrEF
*Statins*							
CORONA (2007)^118^	Chronic HFrEF and IHD >60 years,	Rosuvastatin vs. placebo	5011	33	CVM, MI, or CVA.	No difference.	Only HFrEF
GISSI-HF (2008)^119^	Chronic HF	Rosuvastatin vs. placebo	4574	9	ACM	No difference	Only 10% of patients had LVEF >40%. Sub-optimal compliance to treatment.
*Nitric oxide signalling promoters*							
RELAX (2013)^24^	Chronic HF (LVEF ≥50%, elevated NT-proBNP or filling pressures).	Sildenafil vs. placebo	216	6	Change in peak VO_2_ after 24 weeks.	No difference.	
NEAT-HFpEF (2015)^120^	Chronic HFpEF	Isosorbide mononitrate vs. placebo	110	3	Daily activity level assessed by accelerometry	No difference.	Small trial. Possible induction of nitrate tolerance.
SOCRATES-PRESERVED (2017)^121^	HF with recent exacerbation LVEF >45%	Vericiguat vs. placebo	477	3	Change from in NT-proBNP and LAV.	No difference.	Short treatment duration.
INDIE-HFpEF (2018)^122^	Chronic HF LVEF ≥50% Peak VO_2_ <75% predicted	Inhaled inorganic nitrite vs. placebo	105	3	Peak VO_2_	No difference.	
VICTORIA^a^ (2020)^123^	Chronic HF (NYHA II–IV, LVEF < 45%)	Vericiguat vs. placebo	5050	11	CVM or HF hosp.	35.5% on vericiguat vs. 38.5% on placebo (0.90: 0.82–0.98, *P* = 0.02). HF hosp.: 27.4 vs. 29.6% (0.90: 0.81–1.00). CVM: 16.4 vs. 17.5% (0.93: 0.81–1.06).	Only HFrEF
VITALITY-HFpEF (2020)^124^	Chronic HF LVEF ≥45%	Vericiguat vs. placebo	789	6	KCCQ	No difference.	
*Colchicine*							
Deftereos et al. (2014)^125^	Chronic HF Mean LVEF: 28%	Colchicine vs. placebo	267	6	NYHA	No difference. No difference.	Single-center study. `Only HFrEF.
*Anti-IL**-**1*							
D-HART^a^ (2014)^126^	Chronic HFpEF and CRP >2 mg/L.	Anakinra vs. placebo	12	1	Peak VO_2_	↑ Peak VO_2_ (+1.2 mL/kg/min, *P* = 0.009) and ↓ in CRP (−74%, *P* = 0.006).	Single centre?? Small sample size and short FU.
D-HART2 (2018)^127^	Chronic HFpEF and CRP >2 mg/L	Anakinra vs. placebo	31	3	Peak VO_2_; VE/VCO_2_ slope	No difference.	Small sample size and short FU.
REDHART (2017)^128^	Recent HF hosp. LVEF <50% CRP >2 mg/L.	Anakinra vs. placebo	60	3	Peak VO_2_	No effect after 2 weeks; patients treated for 12 weeks had ↑ in peak VO_2_ (*P* = 0.009).	Small sample size and short FU.
CANTOS-VO2^a^ (2018)^129^	Chronic HF (LVEF <50%) with prior MI and hs-CRP ≥2 mg/L.	Canakinumab vs. placebo	15	12	Peak VO2 and LVEF	Within group analysis: canakinumab ↑ in peak VO2 (*P* = 0.023) and LVEF (*P* = 0.012). No changes on placebo.	Single-centre substudy. Small sample size. Within group analyses.
CANTOS-HF pre-planned subanalysis^a^ (2019)^106^	Chronic HF with prior MI and hs-CRP ≥2 mg/L.	Canakinumab vs. placebo	385	444	Time to first HF hosp.	50 mg (1.04: 0.79–1.36); 150 mg (0.86: 0.65–1.13); 300 mg (0.76: 0.57–1.01, *P* for trend =0.025).	No data on LVEF.
*SGLT2 inhibitors*							
DAPA-HF^a^ (2019)^130^	HFrEF	Dapagliflozin vs. placebo	4744	18.2	Worsening HF or CVM	16.3% on dapagliflozin vs. 21.2% on placebo (0.74: 0.65–0.85, *P* < 0.001). Worsening HF: 10.0% vs. 13.7% (0.70: 0.59–0.83). CVM: 9.6% vs. 11.5% (0.82: 0.69–0.98). Effects similar in presence or absence of T2DM.	Only HFrEF
EMPEROR-Reduced^a^ (2020)^131^	HFrEF	Empagliflozin vs. placebo	3730	16	CVM or HF hosp.	19.4% on empagliflozin vs. 24.7% on placebo (0.75: 0.65–0.86, *P* < 0.001). CVM: 10 vs. 10.8% (0.92: 0.75–1.12). HF hosp.: 13.2 vs. 18.3% (0.70: 0.58–0.85, *P* < 0.001). Effects similar in presence or absence of T2DM. Safety: Uncomplicated genital tract infection more frequent with empagliflozin.	Only HFrEF
EMPEROR-Preserved^a^ (2021)^10^	HF (NYHA II–IV, LVEF >40%)	Empagliflozin vs. placebo	5988	26.2	CVM or HF hosp.	13.8% on empagliflozin vs. 17.1% on placebo (0.79: 0.69–0.90, *P* < 0.001). CVM: 7.3 vs. 8.2% (0.91: 0.76–1.06). HF hosp.: 8.6 vs. 11.8% (0.71: 0.60–0.83). Effects similar in presence or absence of T2DM. Safety: Uncomplicated genital and urinary tract infections and hypotension more frequent with empagliflozin.	
SOLOIST-WHF^a^ (2021)^132^	T2DM recently hospitalized for HF	Sotagliflozin vs. placebo	1222	9	CVM or urgent visits for HF	51.0 per 100 patient-years on sotagliflozin vs. 76.3 on placebo (0.67: 0.52–0.85, *P* < 0.001). CVM: 10.6 vs. 12.5 (0.84: 0.58–1.22).	Few patients with HFpEF (∼80% of the population had LVEF <50%).
DELIVER^a^ (2022)^11^	HF (NYHA II–IV, LVEF >40%)	Dapagliflozin vs. placebo	6263		CVM, HF hosp, or urgent HF visits	To be published.	

Results are presented as (hazard ratio: 95% confidence interval, *P*-value). 6MWT, six minutes walking test; ACS, acute coronary syndrome; CAD, coronary artery disease; CI, confidence interval; CITP, collagen Type 1 C-terminal telopeptide; CKD, chronic kidney disease; CVM, cardiovascular mortality; EAT, epicardial adipose tissue; EF, ejection fraction; eGFR, estimated glomerular filtration rate; HF, heart failure; HFpEF, heart failure with preserved ejection fraction; HR, hazard ratio; hs-CRP, high-sensitivity C-reactive protein; IL-6, interleukin-6; IQR, interquartile range; IV, intravenous; KCCQ, Kansas City Cardiomyopathy Questionnaire; LAV, left atrial volume; LVEF, left ventricular ejection fraction; MI, myocardial infarction; NT-proBNP; aminoterminal-pro-brain natriuretic peptide; NYHA, New York Heart Association; PICP, procollagen Type 1 C-terminal propeptide; PIIINP, procollagen Type III N-terminal propeptide; PUFA, polyunsaturated fatty acids; QoL, quality of life; T2DM, Type 2 diabetes mellitus; VE/VCO_2_, minute ventilation-carbon dioxide production; VO_2_, volume of oxygen consumption.

The trial met the primary endpoints.

### 4.1 Statins

This drug class has several anti-inflammatory properties, including induction of endothelial NO synthase, inhibition of adhesion molecules expression, and reduction of immune cells chemotaxis.^133^ Administration of rosuvastatin is associated with a reduction in hsCRP.^134^ Nevertheless, in patients with HF, the effect of statins on disease progression and death remains uncertain.^118^ In the GISSI-HF trial, rosuvastatin showed no impact on time to death or admission to hospital for cardiovascular reasons in HF patients, irrespective of LVEF; however, only 10% of those enrolled had LVEF >40%.^119^ Interestingly, in the same trial, administration of *n*-3 polyunsaturated fatty acids reduced cardiovascular events by a small amount compared with placebo.^135^ In the CORONA trial, patients in the lowest tertile of aminoterminal-pro-brain natriuretic peptide (NT-proBNP) (roughly <1000 ng/L) had less severe disease and a better prognosis but appeared to benefit from a statin.^136^ Further analyses of the Heart Protection Study confirmed this finding, showing that as NT-proBNP and absolute cardiovascular risk increase, the relative risk reduction with statins shrinks, reaching a point where statin therapy is futile.^137^ A recent collaborative meta-analysis of unpublished data from major primary and secondary prevention RCTs showed that statins modestly reduced the risks of non-fatal HF hospitalization but not HF death.^138^ However, the authors did not have data on LVEF; relatively few were likely to have had HFpEF.

### 4.2 Nitric oxide signalling pathway

NO is an intercellular messenger synthesized and released into the endothelial cells by NO synthases while converting arginine into citrulline. Impaired NO signalling is one of the cardinal features of endothelial dysfunction and atherosclerosis,^139^ as NO inhibits platelet aggregation and promotes vascular smooth muscle cell relaxation.^140^ NO regulates myocardial stiffness and diastolic function in healthy myocardium through the cGMP-protein kinase G pathway.^16,141,142^ Moreover, NO can also balance the functional activity, growth, and death of many immune and inflammatory cell types, including macrophages, T lymphocytes, antigen-presenting cells, mast cells, neutrophils, and natural killer cells.^143^ NO signalling could represent a therapeutic target for myocardial stiffness and inflammation, which are typical stigmata of HFpEF. In an inflammatory milieu, NO bioavailability is reduced by reactive oxygen species^142^ and the inactivation of endothelial NO synthase.^144^ However, in the RELAX trial, 216 patients with chronic HFpEF were randomized to a phosphodiesterase-5 inhibitor known to prolong NO half-life (sildenafil) or placebo. Sildenafil did not improve exercise performance.^24^ Likewise, in the NEAT-HFpEF^120^ and INDIE-HFpEF^122^ trials, nitrates/nitrites failed to improve daily activity level and exercise capacity in patients with HFpEF. Cimlanod, a nitroxyl donor, has been studied in patients with HFrEF in the STAND-UP AHF trial: compared with placebo, this drug led to decreased NT-proBNP plasma concentrations during infusion.^145^ In patients with chronic HFrEF, cimlanod showed haemodynamic effects similar to those of nitroglycerin, i.e. venodilatation and preload reduction without additional inotropic or lusitropic effects.^146^ Ongoing trials of cimlanod will further define its potential role in the treatment of HF.

Vericiguat, an oral soluble guanylate cyclase stimulator, enhances the cGMP pathway by acting synergistically with NO. For patients with HFrEF, the VICTORIA trial suggested a reduction in morbidity and mortality, except in those with a very high NT-proBNP (>5314 ng/L) who appeared to be harmed.^123^ However, for patients with HFpEF (SOCRATES-PRESERVED, *n* = 477 and VITALITY-HFpEF, *n* = 789), vericiguat failed to reduce plasma NT-proBNP concentrations, left atrial volume, or physical activity.^121,124^

### 4.3 Colchicine

Colchicine interferes with cytosolic microtubule assembly inhibiting immune cells chemotaxis and cytokines secretion,^147^ decreasing neutrophil L-selectin expression, thereby inhibiting diapedesis^148^ and inhibiting activation of the NLRP3 inflammasome, which is indirectly responsible for the cleavage of pro-IL-1β to active IL-1β.^149^ Colchicine has been extensively studied with encouraging findings in patients with IHD^96–99^ but with no apparent benefit for patients with chronic HFrEF.^125^ Two ongoing trials could provide valuable insights into the use of colchicine for HFpEF: the COLCOT-T2D will recruit 10 000 patients with T2DM but without known CAD and evaluate whether colchicine reduces cardiovascular risk and progression to HF; the COLpEF (NCT04857931) will enrol 426 patients with HFpEF to assess the effects of colchicine on hsCRP concentrations (primary outcome), symptoms, and other secondary outcomes.

### 4.4 Anti-IL-1

More than 20 years ago, Ridker *et al*.^150,151^ described the significant association of serum concentrations of inflammatory biomarkers (e.g. IL-6 and CRP) with an increased risk of cardiovascular events. Recently, IL-6 has been associated with new-onset HFpEF in community-dwelling individuals.^152^ IL-1β is a key determinant of IL-6 production and has adverse effects on myocardial function in animal models, impairing systolic function, interfering with mitochondrial energy production and uncoupling β-adrenergic receptors, and L-type calcium channels.^153^

Canakinumab is a human monoclonal antibody targeting IL-1β, approved for treating many auto-immune diseases.^154^ In the CANTOS trial, 10 061 patients with a history of MI and hsCRP ≥2 mg/L were randomized to receive canakinumab in three different doses or placebo. After a median follow-up of 4 years, patients treated with the 150 mg and 300 mg dose had a lower incidence of the primary endpoint, a composite of non-fatal MI, non-fatal stroke, or cardiovascular death.^105^ In a prespecified analysis, the use of canakinumab led to a lower incidence of HF hospitalization and HF-related mortality; patients who achieved a hsCRP of <2 mg/L with treatment appeared to derive more benefit.^106^ However, there were no data on LVEF, precluding an analysis by LVEF phenotype.

Anakinra is a recombinant antagonist of the IL-1 receptor, developed as a disease-modifying intervention for rheumatoid arthritis.^155^ The VCU-ART-3 trial enrolled 99 patients admitted for ST-elevation MI with a mean LVEF of 51%; anakinra reduced hsCRP compared with placebo.^107^ The D-HART enrolled 12 patients with HFpEF (median BNP 32 pg/mL) and hsCRP >2 mg/L were assigned to receive anakinra vs. matching placebo. After 14 days, patients assigned to anakinra had a lower hsCRP and greater peak VO_2_.^126^ However, the subsequent D-HART2, which included 32 obese patients with HFpEF and hsCRP >2 mg/L (median NT-proBNP 98 ng/L and 166 ng/L if assigned to placebo and anakinra, respectively), did not confirm an effect on VO_2_, but anakinra reduced hsCRP and NT-proBNP concentrations after 4 weeks.^127^

### 4.5 Sodium-glucose cotransporter-2 inhibitors

SGLT2i, or gliflozins, inhibit the kidney reabsorption of glucose but also appear to have anti-inflammatory effects.^156^ They were primarily developed to treat patients with T2DM, but recent landmark RCTs of HFrEF demonstrated that SGLT2i also reduce HF hospitalizations and cardiovascular death, whether or not the patients have T2DM.^130,131,157^ In the EMPEROR-Preserved trial, nearly 6000 patients with chronic HF and a LVEF >40% were randomized to empagliflozin or placebo. Empagliflozin reduced the incidence of the composite primary endpoint (hospitalization for HF or cardiovascular death), mostly due to a reduction in hospitalization. Haematocrit increased, NT-proBNP fell, and the decline in the estimated glomerular filtration rate was slowed. The benefit was observed for both HFmrEF (LVEF 40–49%) and HFpEF (LVEF ≥50%) regardless of a diagnosis of T2DM.^10^ The ongoing DELIVER trial will assess the effects of another SGLT2i, dapagliflozin, in HFpEF, probably establishing this class of drugs as a cornerstone for treating HFpEF.^158,159^

SGLT2i cause a diuresis, resulting in a reduction in plasma volume and interstitial fluid, an increase in haematocrit and a reduction in body weight. Longer-term, SGLT2i might cause a further rise in haematocrit due to stimulation of erythropoietin and improved iron absorption and further loss in weight due to glycosuria and glucose wasting. However, diverse other potential mechanisms have been proposed, including an anti-inflammatory effect, reduction in oxidative stress and fibrosis, reduced deposition of advanced-glycation end-products, inhibition of the sodium/hydrogen exchanger-1 expressed on cardiomyocyte sarcolemma, and an increase in ketone body production as an energy substrate.^160–167^ Applying artificial intelligence to a cohort of patients with HFpEF, Bayes-Genis *et al*.^168^ recently proposed that SGLT2i act at a molecular level, reducing systemic inflammation by lowering the plasma concentration of NO synthase Type 2 and NLRP3 inflammasome. Improvement in cardiac function with administration of empagliflozin to nondiabetic patients with HFrEF has been linked to a reduction in serum markers of inflammation and EAT volume.^169,170^

### 4.6 Anticoagulants

Activation of inflammatory pathways induces microvascular dysfunction and increases the risk of thrombotic events contributing to the progression of HF.^171^ The generation of thrombin can amplify the effects of other stimuli on inflammatory pathways, which might be ameliorated by reducing its production. The COMPASS trial randomized >27 000 patients with CAD in sinus rhythm to a combination of rivaroxaban 2.5 mg bd and aspirin compared with aspirin and rivaroxaban alone. The combination reduced cardiovascular events and mortality.^172^ The overall result was driven by a large benefit in a subgroup of 4250 patients with HFpEF and mild symptoms;^9^ patients with severe symptoms had been excluded. In contrast, the COMMANDER-HF, conducted in patients with HFrEF, CAD and in sinus rhythm who had a recent decompensation, showed no improvement in the primary endpoint (all-cause mortality, MI, or stroke) with low-dose rivaroxaban vs. placebo.^173^ Overall, these data suggest that the efficacy of this intervention, as for many others, depends on the patient profile. In patients with mild, stable HFpEF (and probably HFrEF), low-dose rivaroxaban may be rather effective, but in patients with more severe HF the dose is either too low or too late because other factors are driving progression.^174^

### 4.7 Influenza and COVID vaccination

Observational studies suggest that influenza vaccination may reduce mortality in patients with HF, and guidelines recommend considering this intervention in patients with HF.^1,175,176^ In a recent double-blind RCT including 2571 patients with a recent MI or severe CAD, influenza vaccination reduced the risk of all-cause mortality, MI or stent thrombosis after 12 months; benefits appeared very early, suggesting a therapeutic effect on the post-MI inflammatory phase.^108^ Unfortunately, the study was terminated prematurely due to the COVID-19 pandemic, but an observational study on >7000 patients with HF showed that COVID-19 vaccination was associated with a substantial reduction in all-cause hospitalization rates and mortality, irrespective of LVEF.^177^ RCTs are ongoing in patients with recent MI or HF.^178^

## 5. Emerging anti-inflammatory targets with potential benefit in HFpEF

HFpEF is associated with the activation of many inflammatory pathways; whether any of these are therapeutic targets is uncertain (*Table 2*).

### 5.1 Anti-IL-6

Recent data suggest cross-talk between IL-1 and IL-6 signalling pathways in HF,^106,179^ leading to increased hepatic CRP production.^52^ Elevated plasma IL-6 concentrations are a hallmark of persistent low-level ‘sterile’ inflammation related to unhealthy ageing, which is characterized by an augmented risk of metabolic and CVD (‘inflamm-ageing’).^180,181^ Indeed, IL-6 can increase vascular smooth muscle cell stiffness and mitochondrial dysfunction, which explains the link between inflammation and impaired vascular function.^52,182^ Interestingly, Tet-2-mutated macrophages secrete higher amounts of IL-6, and these mutations are frequently seen in clonal haematopoiesis of indeterminate potential (CHIP), which is associated with an increased risk of cardiovascular events. In the BIOSTAT-HF study, more than half of the enrolled population had elevated serum concentrations of IL-6, associated with higher NT-proBNP and TNF-α, more iron deficiency, and poorer cardiovascular outcomes.^183^ Moreover, IL-6 administration in animal models was associated with myocardial hypertrophy and fibrosis, promoting diastolic dysfunction.^184^ Thus, IL-6 blockade might reduce the cardiovascular burden in HFpEF, but robust data are lacking.^182^ A subanalysis of the CANTOS trial showed the beneficial effects of canakinumab were more pronounced in those who achieved on-treatment IL-6 concentrations below the study median value of 1.65 ng/L.^179^ In patients with rheumatoid arthritis without CVD, inhibition of IL-6 receptor with tocilizumab was associated with improved LV systolic function and reduced LV mass.^185^ In the ASSAIL-MI trial, tocilizumab increased myocardial salvage as measured by CMR in patients with acute ST-segment elevation MI.^186^ Ziltivekimab, a fully human monoclonal antibody directed against the IL-6 ligand, reduced biomarkers of inflammation and thrombosis among patients with high cardiovascular risk, elevated hsCRP and CKD^187^; a RCT is ongoing to evaluate its clinical value in patients with CV and renal disease (NCT05021835).

### 5.2 C–C chemokine receptor Type 2 modulation

In mouse models, two subsets of cardiac macrophages can be identified according to the surface expression of C–C chemokine receptor 2 (CCR2). Tissue-resident CCR2^–^ macrophages are the most represented subset in a normal heart, showing many cardioprotective properties, like promoting tissue regeneration and coronary angiogenesis. Conversely, only a small amount of inflammatory monocyte-derived CCR2^+^ macrophages is present in healthy mice. The latter initiate inflammation because they can induce neutrophil and monocyte migration into damaged tissues. Mice that developed chronic HF following coronary artery ligation had an increase in CCR2^+^/CCR2^–^ ratio^188^; findings subsequently confirmed in human hearts. CCR2 + macrophage abundance was associated with LV remodelling and more advanced systolic dysfunction in myocardial specimens obtained from HFrEF patients who underwent LV assist device implantation.^189^ An intense proliferation of CCR2^+^ macrophages has also been described in murine models of pressure overload obtained by aortic constriction.^190^ CCR2 modulation could represent a potential target to reduce inflammation and block the development of HFpEF: in murine models of angiotensin-II-induced HFpEF, inhibition of CCR2^+^ macrophages improved diastolic function,^191^ while in humans, the anti-CCR2 humanized monoclonal antibody MLN1202 (plozalizumab) reduced hsCRP in 112 individuals with CV risk factors.^192^ Ongoing exploratory trials targeting CCR2 in different diseases (e.g. T2DM and COPD) could hopefully pave the way to study this therapy in the whole HF spectrum.^12^

### 5.3 Immunomodulation

The systemic inflammatory state can be reduced by targeting cytokine pathways and a generalized modulation of the immune system activity. Cardiosphere-derived cells are a population of cardiac progenitor cells with prominent anti-inflammatory properties. In a rat model of hypertensive HFpEF, intracoronary treatment with cardiosphere-derived cells reduced serum concentrations of inflammatory cytokines, improved diastolic function, and decreased myocardial fibrosis, despite persistent hypertension.^193^ The ongoing REGRESS-HFpEF trial (NCT02941705) will assess whether intracoronary administration of cardiosphere-derived cells can reduce pro-inflammatory and pro-fibrotic signalling, as well as improve functional status and haemodynamics in patients with HFpEF.

### 5.4 Exercise training

Impaired exercise capacity is a hallmark of HFpEF,^194^ which is likely to be multifactorial, including cardiac dysfunction, skeletal muscle deconditioning, obesity, co-existent lung or joint disease, and psychological factors.^195^ In patients with HFpEF, endurance exercise training was associated with increased functional capacity (as assessed by peak VO_2_) and quality of life but no improvement in endothelial function or arterial stiffness.^196,197^ Physical training reduces systemic vascular resistance^198^ and increases skeletal muscle perfusion, peripheral oxygen utilization, and mitochondrial function.^199,200^ These effects further modulate inflammatory and oxidative processes^201^ that may benefit patients with HFpEF.^202,203^

### 5.5 Epigenetics regulators

The term ‘epigenetics’ encompasses the changes that affect gene activity and expression without involving alterations in the genome (DNA sequence).^204^ The administration of epigenetics regulators in animal models of hypertensive cardiomyopathy reduced TNF-α concentrations and interstitial myocardial fibrosis.^205^ The BETonMACE trial enrolled 2425 patients with T2DM up to 3 months after an acute coronary syndrome: participants were randomized to placebo and apabetalone, an inhibitor of bromodomain and extra-terminal motif proteins, which are epigenetic modulators of inflammation, thrombogenesis, and lipoprotein metabolism implicated in atherothrombosis.^206^ Although the trial missed its primary endpoint (MI, stroke, or cardiovascular death), in a prespecified secondary analysis, treatment with apabetalone was associated with a lower incidence of HF hospitalization than placebo^110^ {hospitalization for HF or cardiovascular death [5.7 vs. 7.8%, hazard ratio 0.72 (95% confidence interval 0.53–0.98), *P* = 0.04]}. Unfortunately, there were no data about LVEF; thus, further RCTs are needed to evaluate whether epigenetic modulators represent another promising therapeutic approach to preventing and treating HFpEF.

Micro-RNA (miRNA) are small, non-coding RNAs involved in the RNA-induced silencing complex, which binds messenger RNA either inducing its degradation or inhibiting its translation at the ribosomal level.^207^ A single miRNA can act as an epigenetic modulator, regulating the expression of hundreds of different mRNAs without modifying the gene sequences.^208^ Several cardiovascular conditions seem to be associated with specific miRNAs: for example, serum concentrations of miR-210 and miR-1 correlate with symptom severity in HF.^209,210^ Furthermore, inhibition of miRNA-21 prevented the development of HFpEF in an experimental model.^211^

### 5.6 Myeloperoxidase inhibitors

Extracellular deposition of granulocyte-derived myeloperoxidase (MPO) can cause oxidative stress, leading to microvascular dysfunction, inflammation, tissue damage, and fibrosis. A novel MPO inhibitor (AZD4831) reduced inflammation and improved microvascular function in preclinical models.^212^ Target engagement and safety of AZD4831 have been tested in a Phase 2a study of HFpEF (NCT03756285), supporting further development.

### 5.7 Adrecizumab

Adrenomedullin is a peptide hormone synthesized by endothelial and vascular smooth muscle cells. Its production is stimulated by volume overload to maintain endothelial barrier function, while the disruption of the adrenomedullin system results in vascular leakage and systemic and pulmonary oedema. Adrenomedullin is markedly elevated in patients with sepsis and in patients with acute HF, probably as a compensatory mechanism against fluid overload and tissue congestion.^213^ Adrecizumab is a monoclonal, non-neutralizing antibody that stabilizes adrenomedullin, ‘trapping’ it in the circulation without blocking adrenomedullin receptor signalling. In addition, adrecizumab translocates adrenomedullin from the tissue into the circulation. In animal models of systemic inflammation and septic shock, adrecizumab-induced increases in plasma adrenomedullin improved haemodynamics, renal function, and reduced markers of inflammation.^213^ A Phase II proof of concept study in patients hospitalized for acute HF is ongoing (NCT04252937).

### 5.8 Epicardial adipose tissue

In patients with HFpEF, EAT accumulation is often marked, and it promotes haemodynamic derangements,^31–33^ altered adipogenesis by secretion of pro-inflammatory and pro-atherogenic adipokines,^34^ and an adverse prognosis.^81^ Noteworthy, increased biventricular hypertrophy and EAT exacerbate pericardial restraint in HFpEF, resulting in higher LV filling pressure to achieve a given transmural pressure, particularly during exercise.^32^ Enhanced pericardial restraint in patients with HFpEF partially explains the lower concentration of natriuretic peptides observed in this cohort, showing pathophysiology similar to that observed in constrictive pericarditis.

Thus, in selected HFpEF patients, non-pharmaceutical interventions (e.g. exercise, diet, or bariatric surgery)^214^ or pharmacological therapies (e.g. SGLT2i and GLP-1 agonists)^35^ targeting excessive EAT accumulation might decrease inflammation and potentially provide meaningful clinical benefits.

## 6. Conclusions

Subclinical inflammation is common in patients with HFpEF, regardless of underlying aetiology and associated comorbidities. A deeper understanding and detailed characterization of inflammatory mechanisms responsible for disease onset and progression may lead to new therapeutic opportunities to improve the well-being and outcomes of those with or at risk of developing HFpEF.

## Data Availability

There are no new data associated with this article.
